# Development of an Activity-Dependent Epidural Stimulation System in Freely Moving Spinal Cord Injured Rats: A Proof of Concept Study

**DOI:** 10.3389/fnins.2018.00472

**Published:** 2018-07-23

**Authors:** Avi Rascoe, Pawan Sharma, Prithvi K. Shah

**Affiliations:** ^1^Division of Rehabilitation Sciences, Department of Physical Therapy, School of Health Technology and Management, Stony Brook University, Stony Brook, NY, United States; ^2^Department of Neurobiology and Behavior, Stony Brook University, Stony Brook, NY, United States

**Keywords:** activity-dependent electrical stimulation, closed-loop stimulation, epidural stimulation, cervical spinal cord injury, hand function, rats

## Abstract

**Purpose:** Extensive pre-clinical and clinical experimentation has yielded data on the robustness and versatility of epidural stimulation (ES) strategies to activate spinal neural circuitry to produce functional benefits. Increasing studies are now reporting that closed-loop electrical stimulation delivery methods significantly enhance the neuromodulation effects of stimulation, to in turn, improve physiological outcomes of the intervention. No studies have yet explored the feasibility and usage of closed-loop systems to neuromodulate the cervical spinal cord using ES.

**Methods:** We developed an activity-dependent system that utilizes electromyography (EMG) activity to trigger epidural stimulation (tES) of the cervical spinal cord in awake, freely moving rats. Experiments were performed on rats that were implanted with chronic forelimb EMG and cervical epidural implants, with (*n* = 7) and without (*n* = 2) a complete C4 spinal hemisection.

**Results:** Our results show that the EMG triggered activity-dependent system can be reliably applied and reproduced for: (i) stimulating multiple rats simultaneously throughout the night during free home-cage activity and (ii) use as a mobile system for testing and training during various short-term behavioral testing conditions. The system was able to consistently generate stimulation pulse trains in response to attempted EMG activity that crossed a user-defined threshold in all rats for all experiments, including the overnight experiments that lasts for 7 h/session for 6 days/week through the 3-month period.

**Conclusion:** The developed closed-loop system can be considered to represent a class of bidirectional neural prostheses via a circuit that enables two-way interactions between neural activity (real-time processing of EMG activity) and external devices (such as a stimulator). It can operate autonomously for extended periods of time in unrestrained rats, allowing its use as a long-term therapeutic tool. It can also enable us to study the long-term physiological effects of incorporating electrical stimulation techniques into the nervous system. The system can also be experimented for connecting several neural systems into a *Brainet* by combining neural signals from multiple rats dynamically and in real-time so as to enhance motor performance. Studies are ongoing in our laboratory to test the usefulness of this system in the recovery of hand function after cervical spinal cord injuries.

## Introduction

Electrical epidural stimulation (ES) of the spinal cord has gained increased attention as a successful neuromodulatory strategy for functional recovery after a severe spinal cord injury (SCI) in humans. After prescribed delivery and hence *training* with ES, patients with a complete SCI have demonstrated restoration of critical body functions including voluntary leg (Carhart et al., 2004; Angeli et al., 2014; Grahn et al., 2017) and arm (Lu et al., 2016) movements, independent standing (Harkema et al., 2011; Rejc et al., 2015) posture, bladder, bowel (Harkema et al., 2011; Angeli et al., 2014), and physiological cough performance (DiMarco et al., 2009, 2014). It is now becoming clear that spinal ES not only activates peripheral afferents, dorsal columns and motoneurons (Capogrosso et al., 2013; Minassian et al., 2016), but also has a neuromodulatory effect on a variety of segmental spinal interneurons (Gad et al., 2013; Taccola et al., 2017). Consequently, this results in drastic alterations in the excitability state of spinal neuronal circuits as well as of spared descending supraspinal connections to a level at which spinal circuitries can generate voluntary functional motor output.

Two major advancements in electrical stimulation strategies portend the continued success of ES as a crucial tool for neuromodulation in the field of SCI neurorehabilitation: first, multi-site stimulation of the spinal cord has gained increased attention because of its ability to spatially and functionally activate wide and discrete neuronal populations to synergistically influence and modulate the excitability of sensorimotor pathways for an effective motor output, more so than single site stimulation of localized individual segments (Angeli et al., 2014; Gerasimenko et al., 2015; Sayenko et al., 2015; Shah et al., 2016). As such, differential excitation of local spinal segments seems to be the key to neuromodulate appropriate neuronal networks for functionally beneficial outcomes.

Second, the advent of closed-loop electrical stimulation systems has become an attractive option in neurorehabilitation. Closed-loop is defined as the “delivery of any form of therapy, either to a target or systemically, exclusively in response to a specific cue or command” (Osorio et al., 2001). Closed-loop electrical stimulation techniques involve delivery of stimulation in response to an endogenous neural drive, such as a neural or behavioral event, to permit modulation of active neurological signals without experimenter input. In biological studies, the term closed-loop is also used for a technique in which bio-signals from the subject determine the timing of stimulus delivery and stimulation delivery is continually adapted in response to physiological changes (Sun and Morrell, 2014; Krook-Magnuson et al., 2015; McPherson et al., 2015). This is in contrast to studies that deliver open-loop stimulation protocols in which the stimulus is delivered regardless of ongoing activity and according to a predefined offline script (Gerasimenko et al., 2008; Shah et al., 2016). Indeed, increasing studies are now reporting that such closed-loop electrical stimulation delivery methods that control the timing of stimulation delivery so as to pair it with an ongoing neural activity, significantly enhance the neuromodulation effects of stimulation, to in turn, improve physiological outcomes of the intervention (Fountas et al., 2005; Rolston et al., 2009; Venkatraman et al., 2009; McPherson et al., 2015; Zareen et al., 2017). The purported mechanism is a direct test of Hebb’s postulate (Hebb, 1949), showing that natural patterns of neuronal firing can lead to input-specific plasticity when paired with appropriate postsynaptic depolarization during normal behavior (Jackson et al., 2006a; McPherson et al., 2015). As such, it is not surprising that stimulation that is delivered precisely at the initiation of activity leads to potentiation of an already active circuitry to produce functional benefits (McPherson et al., 2015; Mercier et al., 2016). Additionally, there is also evidence for the retention of long term functional reorganization of neuronal circuitry with closed-loop stimulation (Jackson et al., 2006a).

The majority of closed-loop stimulation protocols currently employed are used to stimulate brain structures and are utilized for the treatment of a variety of neurological disorders in humans (Fountas et al., 2005; Little et al., 2013; Krook-Magnuson et al., 2015). Most closed-loop systems also adopt circuitry designs that are complex and expensive, require custom building of neuro-chips, demand tester supervision, not particularly tested for chronic stimulation and/or do not necessarily provide modifiable circuit schematics (Zanos et al., 2011). Although pre-clinical studies utilizing closed-loop stimulation techniques of the spinal cord have just begun to surface (Fuentes et al., 2009; Wenger et al., 2014; Zimmermann and Jackson, 2014; McPherson et al., 2015; Mercier et al., 2016), most of these systems either utilize cortical signals for stimulation (Fuentes et al., 2009), use intraspinal microstimulation techniques (Fitzsimmons et al., 2007; Fuentes et al., 2009; Zimmermann and Jackson, 2014; McPherson et al., 2015; Mercier et al., 2016) or have been tested for ES of the lumbosacral cord (Wenger et al., 2014). Experiments that use kinematics and/or electromyography (EMG) to trigger lumbo-sacral ES in thoracic spinal rats incorporate half an hour of neuromodulatory rehabilitative training sessions under tester supervision (Gad et al., 2012; Wenger et al., 2014). No studies have yet explored the feasibility and usage of closed-loop systems to neuromodulate the cervical spinal cord using ES, nor have demonstrated the effects of chronic ES of the cervical cord during overnight activity.

In the present study, we assemble a closed-loop activity-dependent system capable of delivering low-latency stimuli subsequent to detection of a user-defined bio-signal. Although this system has varied applications, we specifically demonstrate a closed-loop stimulation technique that effectively allows multi-channel ES of the cervical spinal cord triggered by EMG activity (tES) of forelimb muscles in awake, freely moving rats. This application is a preamble to ongoing experiments in our laboratory that aim to recover upper limb sensory-motor function after a cervical SCI in rodents. We present details of the setup with demonstration of its application in rats with and without a cervical hemisection injury. In this proof of concept study, we highlight unique features of the technique and validate the feasibility and reliability of the closed loop system, and delivery of tES (i) during overnight long-term stimulation in the rat’s home-cage during overnight activity (7 h/day, 6 days/week for ∼1–3 months) without tester supervision in multiple rats simultaneously and (ii) during short-term stimulation during a variety of motor activities in controlled experimental conditions.

## Materials and Methods

### Animals and Experimental Design

This study was carried out in accordance with the recommendations of National Institutes of Health Guide for the Care and Use of Laboratory Animals. The protocol was approved by the Stony Brook University Chancellor’s Animal Research Committee (National-Research-Council, 2011).

**FIGURE 1:**
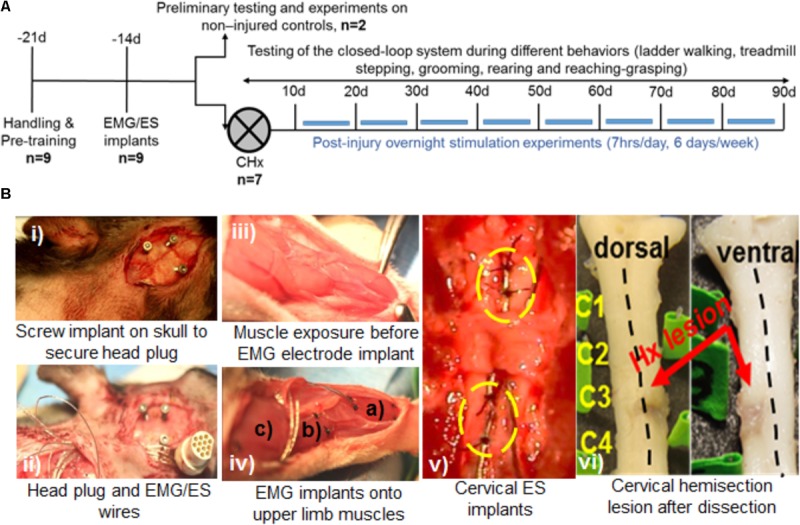
Experimental outline, behavioral and surgical procedures. **(A)** Rats were initially handled prior to all experiments and trained for treadmill stepping and reaching-grasping activities. Later, rats underwent EMG and ES implants and were randomized into two experiments: *Experiment I:* two rats served as non-injured controls and were subjected to several pilot trials for initial testing of the closed-loop ES system in a variety of motor behaviors (open field walking, ladder walking, treadmill stepping, and grooming) and overnight stimulation experiments. *Experiment II:* seven rats received a cervical hemisection injury at the C4 spinal segment (CHx). They were trained with triggered ES (tES) during normal night cage activity for ∼7 h/day, 6 days/week for starting at 10 days after injury. Open field, ladder stepping, rearing, reaching-grasping and grooming tests were performed at multiple time points throughout the study. **(B)** Still photographs of EMG/ES implants and the CHx site. **(i)** Three screw implants on the skull were used to secure the head plug connector. **(ii)** EMG/ES electrode wires are routed subcutaneously underneath the neck region and routed toward the upper back. **(iii)** Exposure of forelimb muscles prior to EMG implantation. **(iv)** Select forelimb muscles [**(a)** flexor digitorum **(b)** pronator **(c)** deltoid] are implanted with EMG electrodes. **(v)** Epidural implants are shown at C6 (upper circle) and C8 (lower circle) spinal segments with two suture knots (black knots within circles) to secure the implants. **(vi)** Gross dorsal and ventral views of the cervical spinal cord shows the CHx lesion site at C4 replaced with scar tissue.

An outline of experimental procedures is shown in **Figure 1A**. A total of nine adult female Sprague-Dawley rats (240–260 g body weight) underwent surgical procedures for chronic EMG and epidural stimulating electrode implantation. Two rats served as non-injured controls and 7 rats were subject to a complete cervical hemisection (CHx) at the C4 spinal segment. We first tested feasibility of implementing the closed-loop system that consisted of EMG triggered ES (tES) in non-injured rats (*n* = 2). Specifically, we tested if the developed system could deliver ES triggered from muscle activity during the rat’s night activity in its home cage with a 7 h tES training protocol (non-mobile setup, described below). The functionality of the closed-loop system to deliver tES was also tested during different motor behaviors (mobile setup, described below). Reliability and validity of the closed-loop system to deliver tES was then assessed by exposing CHx injured rats to tES for both the mobile and non-mobile setups throughout the experimental period for 3 months (*n* = 7).

### Surgeries

All surgeries were performed under aseptic conditions with the rats deeply anesthetized with isoflurane gas (1.0–2.5% via facemask as needed). Surgery was performed with the rats on a heated surgery table (Shor-Line, Kansas City, MO, United States) and maintained at 37°C to maintain body temperature. All incisions were closed in layers using 5.0 Vicryl for the muscle and fascial layers, and staples for the skin. After surgery, the rats were placed in an incubator until fully recovered and administered antibiotics and analgesics as needed for up to 5 days. Thereafter, the rats were housed singly in cages to avoid damaging each others’ head plugs through social interaction. The room was maintained at 26 ± 1°C, 40% humidity and a 12:12 h light:dark cycle with access to food and water *ad libitum*. The cage floors were covered with Alpha-Dri Bedding (Shepard Specialty Papers, Watertown, TN, United States). Pieces of fruit were given during each animal care session. For rats that underwent CHx, the bladders of all rats were expressed manually two times daily for the first 1–3 days until they regained complete bladder control.

#### Electromyography Implants

A small incision was made at the mid-line of the skull. The muscles and fascia were retracted laterally, few superficial cuts were made in the skull with a scalpel (to roughen the skull surface to accommodate dental cement), and the skull was dried thoroughly. One connector with Teflon-coated stainless-steel wires (Omnetics, Part # AS632; Cooner Wire Co., Chatsworth, CA, United States) was attached securely to the skull with screws (**Figure 1B-i**) and dental cement as previously described (Roy et al., 1991; Shah et al., 2012). While the screws hold the head plug in place, the dental cement firmly secures the screw and head plug unit onto the skull, making this ‘head mount’ on the skull a permanent one for use in chronic stimulation experiments. A skin incision was made in the upper-dorsal region of the back and wires from the connector were routed subcutaneously (**Figure 1B-ii**). Two wires were coiled subcutaneously so that they could be easily retrieved for implantation as ES electrodes on the spinal cord at a later time point (see below). Skin and fascial incisions were made to bilaterally expose belly of the deltoid, pronator, flexor digitorum, and extensor digitorum muscles (**Figure 1B-iii**). Muscle identity was verified using direct muscle electrical stimulation. Wires were routed subcutaneously from the back incision to each muscle site. Bipolar intramuscular EMG electrodes were formed and secured into the mid-belly of each muscle as shown in **Figure 1B-iv** and as described previously (Roy et al., 1991). The proximal ends of EMG wires outside the muscle were coiled near each implant site as well as in the mid-back region and placed beneath adjacent fascia. The coiled wires provided room for stress relief during rat movement. After electrode implantation into the muscle, we verified proper placement of the EMG electrodes into the desired muscle by stimulating the muscle via the head plug connector. Approximately 1cm of Teflon coating was stripped at the distal end of an additional wire that served as a reference electrode: this wire was placed subcutaneously on the right side of the vertebral column at the level of the inferior scapular angle.

#### Epidural Implantation

Epidural electrodes were implanted as described previously (Shah et al., 2012, 2016; Alam et al., 2015). Briefly, a longitudinal skin incision was made on the upper back using the C2 and T2 spinous processes as landmarks. Underlying fascia and upper back muscles (trapezius, rhomboid, and splenius) were then retracted with blunt dissection to expose the cervical vertebral column. A partial laminectomy was performed at vertebral levels C4, C5, and C7. Laminectomies at C5 and C7 were adequate to expose spinal cord segments C6 and C8, respectively. Partial laminectomy of C4 vertebra allowed passage of the Teflon-coated stainless-steel wires. These wires were pulled toward the deeper back musculature and then inserted beneath the partial C4 vertebrae and passed epidurally to each partial laminectomy site. One additional wire that served as a reference electrode was placed subcutaneously on the left side of the vertebral column at the level of the inferior scapular angle. A small region (∼1 mm notch) of the Teflon coating was removed from each wire to form the stimulating electrodes that were then secured to the dura at the mid-line of the spinal cord at each site with 8.0 Ethilon sutures (**Figure 1B-v**). The wires were coiled at the exit site from the vertebral column to provide stress relief.

#### Cervical Spinal Cord Hemisection

A dorsal mid-line skin incision was made and the paravertebral muscles and fascia from ∼C2 to T2 vertebral body levels were reflected laterally to expose the vertebrae. A partial laminectomy was performed via removal of the spinous processes and a portion of the lateral bodies of the C3 and C4 vertebrae, effectively exposing the spinal cord. A 30-gauge needle was inserted into the midline of the cord to demarcate the right and half halves of the spinal cord. Micro-scissors were used to transect the right half of the spinal cord at ∼C4. Injury was verified by gently passing a fine glass probe through the hemisection site. Post-mortem, all injuries were verified histologically (**Figure 1B-vi** shows gross lesions, histology data not shown).

### Development of a Closed-Loop ES System

**FIGURE 2:**
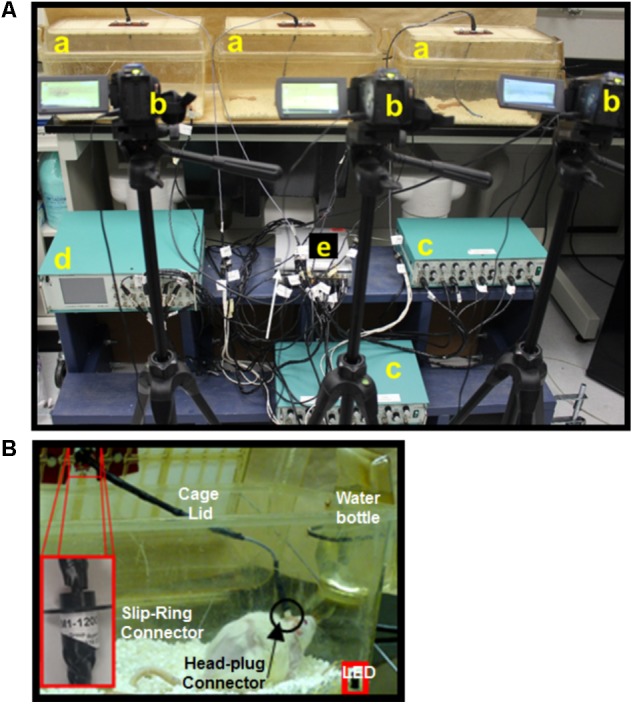
Non-mobile experimental setup for long-term closed-loop spinal epidural stimulation (ES) in multiple rats. **(A)** Picture of the experimental setup showing multiple home cages **(a)**, cameras **(b)**, amplifiers **(c)**, a stimulator **(d)**, and a DAU **(e)**. **(B)** Picture of the rat in its modified home-cage allows full access to water without compromising movement due to the cable. A slip-ring connector (insert) linked to the head plug cable is attached into the cage lid and allows the rat a full 360 degrees of range of motion in the home-cage.

**FIGURE 3:**
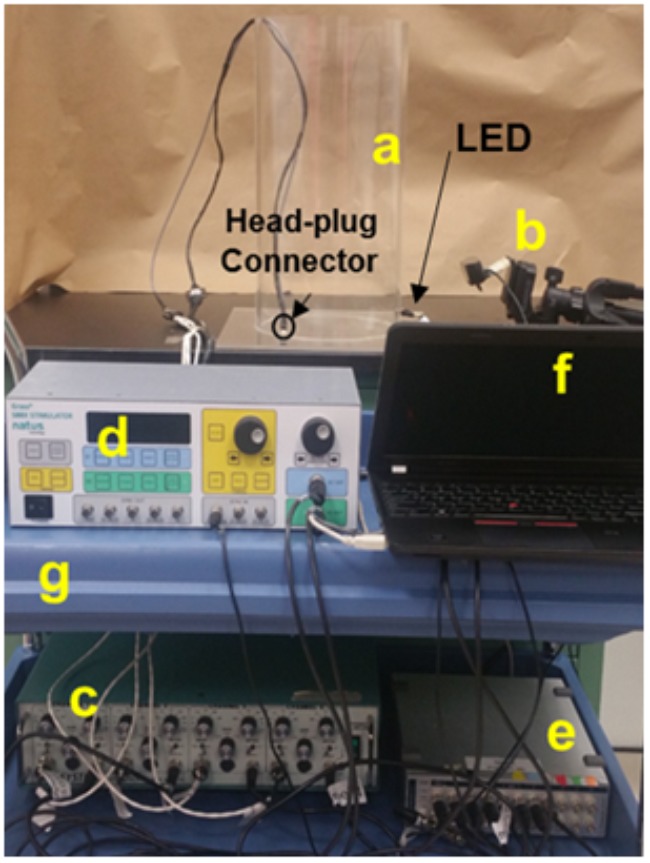
Mobile experimental setup utilizing the closed-loop spinal ES system. Picture of the experimental setup for an individual rat shows an acrylic cylinder used for obtaining EMG data during grooming and rearing behaviors **(a)**, camera **(b)**, amplifier **(c)**, stimulator **(d)**, DAU **(e)**, and laptop **(f)**, all situated on a mobile cart **(g)**. The camera setup is particularly different than the non-mobile experiments and the setup is handy for training and testing in short experiments.

We developed and tested a closed-loop ES system for the cervical cord by integrating key components of an effective closed-loop system (Krook-Magnuson et al., 2015) for use in freely moving rodents: *(i) Input trigger:* provided by salient EMG signals from forelimb muscles implanted with chronic EMG electrodes. *(ii) Real-time processing of the bio-signal to trigger stimulation*: performed by the data acquisition unit (DAU). *(iii) Closed-loop delivery of stimulation*: via chronically implanted cervical epidural electrodes. This loop although does not implement a feedback controlled error correction mechanism by determining the ‘correctness’ of occurrence of that signal, the system is closed-loop in that it utilizes a recurrent biosignal-machine interface wherein real-time neural activity from the subject determines the timing, stimulation frequency and the length of stimulus delivery to the target. Additionally, the stimulation delivery stops once the rat stops to move. This is in contrast to open-loop stimulation strategies that we and others have used previously to continuously and independently deliver stimulation to the subject (Courtine et al., 2009; Shah and Gerasimenko, 2016). Although the hardware assembly may be used for a variety of applications, we demonstrate feasibility and usage of this setup in two experimental settings relevant to our laboratory: Chronic longitudinal experiments that require overnight training with tES for 7 h/day for 6 days/week for ∼3 months (non-mobile setup, **Figure 2**) and experiments aimed at assessment and training in a variety of rehabilitative motor tasks (mobile setup, **Figure 3**). Details of the setups are described in ‘setup for data collection’ below. In both setups, the amplified EMG signal is conveyed from the rat to the DAU, which signals the stimulator for delivery of ES.

#### From the Rat to the Data Acquisition Unit (DAU)

**FIGURE 4:**
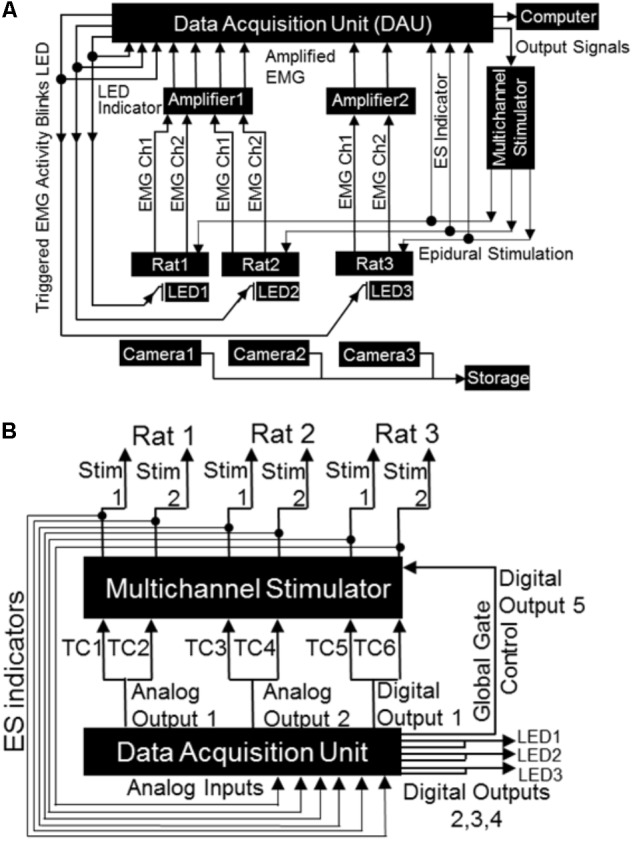
Block diagrams depicting electrical connections of the mobile closed-loop spinal ES system. **(A)** Shown is a setup for a non-mobile closed-loop ES system for three rats. Detected EMG signal (shown are two EMG channels per rat) from implanted electrodes is amplified and sent to the DAU. When the EMG signal crosses a user defined threshold, the DAU triggers the multi-channel stimulator and synchronizing LED. A head plug cable transmits the stimulation pulse to the rat’s spinal cord. The synchronizing LED pulse is visible as a bright light on the camera each time the EMG signal crosses the threshold. Concurrent inputs into the DAU monitors the LED pulse as well as sends output signals to the stimulator. A computer records all inputs that is fed into the DAU (EMG signal, LED pulse, stimulation pulse). An infrared camera records rat activity throughout the long-term night experiments. **(B)** Expanded block diagram from **(A)** above shows detailed connections and ES indicator loops from the DAU to the stimulator. Included are the global gate output control for the stimulator, the LED outputs and LED indicator loops (TC: stimulator trigger input channel).

The built closed-loop circuitry for ES triggered by EMG signal is shown schematically in **Figure 4A**. Twelve pin circular plastic connectors (Part # A22004-001; Omnetics Connector Corp., Minneapolis, MN, United States) implanted on the rat’s skull conveyed EMG signal from the forelimbs to the DAU through an amplifier. Nine of the pins (for EMG) from the mating connector were connected to one end of a 12-connector cable (Part # NMUF 12/30-4046SJ; Cooner Wire Co., Chatsworth, CA, United States). The remaining three pins (for ES) were soldered to three hook-up wires. Ends of these cables/wires were soldered to the rotary end of a slip-ring commutator (Part # 312M1-1200; Orbex Group, Fremont, CA, United States) implanted in the cage lid (**Figure 2B**). Commutators were used to ensure that free movement of rats in the cage is not limited by cable rotations. Non-rotary end of the slip-ring were connected to two D-SUB 9 connectors, one each for EMG and ES. The mating D-SUB connector for the EMG cable were soldered onto the ends of a 3′ input cable (Part #692000; A-M Systems, Sequim, WA, United States) for use with an amplifier (A-M Systems, Sequim, WA, United States). The mating D-SUB connector for the ES cable received input from the stimulator. Note that for the mobile experimental setup, the wires and cables that are soldered to the mating connector were attached directly to the D-SUBs without slip rings. The amplifier settings were as follows: Gain: x1000, Low Cut-off frequency: 10 Hz, High Cut-off frequency: 5 kHz, Notch settings were adjusted to ‘*In.*’ The amplifier was connected to the analog input channels on the DAU (ADI Inc., Colorado Springs, CO, United States).

#### From the DAU to the Stimulator and LEDs

The DAU was connected to the stimulator (for delivery of ES) and to LEDs (for visual syncing of infrared cameras and EMG activity) as follows (**Figure 4B**). Each of the two DAU analog output channels were split so as to send signals to two separate external trigger channels of the stimulator (A-M Systems or Grass S88x Natus Medical Inc., Pleasanton, CA, United States). To create additional output channels (one for a third stimulation trigger for a third rat, one for global gate control and three for LEDs), a single D-SUB 15 cable was custom constructed and outputted from the digital outputs on the DAU. This cable was soldered to a two row DSUB-15F connector and had the following connections: (a) three BNC connectors and LEDs with resistors. The BNC cables connected in-line with the LEDs monitored LED pulses via the DAU. These LED-BNCs were then connected to analog-in channels of the DAU for viewing as separate channels on the software. (b) A stimulator trigger channel (for the third rat), the digital output of which was split similar to the analog output channels. (c) A digital out channel that was used for the stimulator global gate control, which served as a master on/off switch for delivery of stimulation, ultimately controlled by the software. Note that the digital outputs of DAU for the additional rats can be used to expand the setup to include five rats (with two triggering muscles, one DAU, and one stimulator) or to a maximum of eight rats (with two triggering muscle, two DAU and one stimulator) simultaneously, but we demonstrate here a setup for only three rats. For the mobile experimental setup, a single LED was soldered onto one end of a BNC cable and attached to a splitter connected to the analog output of the DAU. The remaining end of the splitter was connected to an analog input channel on the DAU to allow for recording of LED pulses by the DAU. In all cases, the LEDs were placed near the rat’s cage within the field of view of the cameras that captured all motor behavior either for overnight or mobile experiments. An infrared (IR) camera was used for the overnight filming (Bell & Howell, Elite Brands Inc., New York, NY, United States), and a webcam for the mobile experiments.

#### From the Stimulator to the Rat and DAU

Each output channel of the stimulator was split using a BNC splitter. One BNC cable was connected to an analog input channel on the DAU to monitor the outputted voltage from the stimulator and the other connected to a DSUB-9 from where a cable connected with the rat’s head plug on the skull.

#### Software and Stimulation Protocols

All control settings of the closed-loop system were made in either ADI’s Labchart or AM-Systems 3800 software. For the mobile system, ES was directly controlled via the stimulator. The software settings for EMG and ES were as follows.

##### Delivery of a trigger pulse initiated by EMG activity

An EMG threshold for triggering ES was first identified by collecting 3–5 min of baseline EMG data while the rat freely moved around in the cage. In order to prevent the stimulator from delivering multiple trigger outputs during the same EMG event, hysteresis was set to 1–3%, thereby necessitating a decrease in signal to 1–3% of the total dynamic range to generate the subsequent trigger pulse. This value was set depending upon baseline noise levels and amplitude of EMG signals. The triggering pulse width was set to 5 ms for the stimulation and LED triggers. Software settings also allowed for (i) comment placements on a separate digital channel to identify which muscle in each animal triggered the stimulation (ii) automatically start the stimulation after 30 min of baseline data collection, and (iii) obtain additional 30 min of data after the termination of the stimulation period. For the non-injured rats, the deltoid muscle was used to trigger stimulation since it was the first muscle to become activated during most cage activity. Note that for injured rats too, the deltoid muscle EMG threshold continued to trigger ES (since the C4 injury resulted in partial sparing of the muscle). In addition, stimulation was triggered off the wrist extensor muscle, which showed some activity by 10 days post-injury (dpi) in most rats.

##### *Delivery of ES* triggered by onset of EMG activity (tES)

Stimulation on/off was controlled by a digital output channel attached to the global gate of the stimulator (see output signals in **Figure 4B**). With the rat connected to the setup, the stimulation intensity was first identified to elicit the lowest visually detectable upper limb muscle twitching (in both injured and non-injured rats). This was repeated for both channels (C6 and C8) for each rat. In both non-injured and injured rats, we used minimal visible twitching of the upper limb to identify the stimulation threshold. And this measure remained same throughout the study. Note that we were able to detect forelimb muscle twitching in response to ES at 10 dpi. Intensity of stimulation voltage for all experiments was then set at 90% of the obtained motor threshold (ES threshold voltage). Sub-motor threshold stimulation intensities were utilized to avoid interference with voluntary movement, eating, drinking and sleeping activities during the stimulation. In the current experiments, we used voltage stimulation to simulate our previous works (Shah et al., 2012; Alam et al., 2015; Shah and Lavrov, 2017). Note that for experiments when current output is desired, stimulation is possible for up to 10 h, being limited by the battery life of the stimulus isolator units (data not shown). Once the stimulation intensity threshold was set, our software was programmed to deliver this ES threshold voltage only when the EMG amplitude on the desired channel went above a user-defined EMG threshold based off EMG activity during attempted movement (EMG threshold). This EMG threshold was necessary to take into account any baseline electrical noise and avoid unnecessary delivery of ES independent of EMG activity. The software then triggered delivery of ES from onset of EMG activity or EMG spikes within a burst.

**FIGURE 5:**
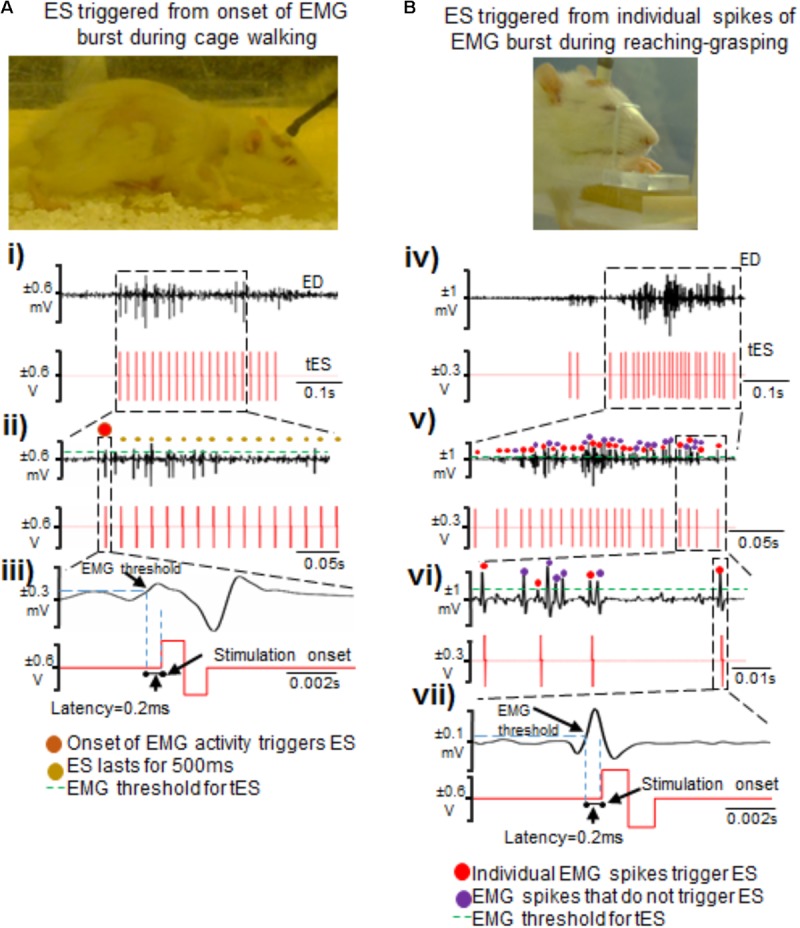
Two methods of triggering ES from EMG signals during attempted activity. **(A)** Shown are standstill images of a rat receiving tES triggered from the onset of the extensor digitorum (ED) muscle bursting activity during walking in the home-cage. **(i)** Single ED burst during walking with corresponding stimulation train. **(ii)** Shown is a zoomed-in view of the EMG burst. The red dot represents the first spike that initiated the train of ES pulses (shown as red biphasic waveform). **(iii)** A single spike along with corresponding ES pulse triggered from that spike is zoomed to demonstrate latency for onset of stimulation pulse after the EMG spike crosses a set threshold. **(B)** Shown are standstill images of a rat receiving tES triggered from individual spikes (EPSPs) in an EMG burst during a reaching-grasping task. **(iv)** Biphasic stimulation pulses are delivered in response to individual spikes in an EMG burst that crosses a user defined threshold. **(v)** Zoomed-in view of the EMG signal shows individual spikes and corresponding stimulation pulses. Red dots are the spikes that triggered tES. Violet dots are the spikes that cross the user-defined threshold, but do not trigger tES because of the preset inter-stimulation interval time. **(vi)** Expanded view of individual spikes that triggered (red dots) or failed to trigger (violet dots) tES. In this example, an inter-stimulation interval is set to 10 ms to prevent stimulation artifacts from retriggering stimulation. **(vii)** Note the latency of ∼0.2 ms from time of spiking activity to delivery of tES, similar to what is seen in **(iii)**.

As proof of principle, we demonstrate two kinds of stimulation parameters using the closed-loop system. First, a 500 ms pulse train at 40 Hz that consisted of biphasic rectangular pulses (200 μs duration) was triggered by onset of EMG burst during free cage activity (**Figures 5A-i–iii**). Stimulation was delivered to the C6 and/or C8 spinal segments with monopolar configurations of C6-Ref+ and C8-Ref+ as described before (Alam et al., 2015; Alam et al., 2017). The length of the train was set to 500 ms at 40 Hz frequency based off an average bursting period of 0.5 ms for most muscles during motor activity. Thus, once a train of ES pulses was delivered in response to initiation of EMG burst activity, subsequent spiking activity from the EMG burst did not generate any new stimulation pulses until the train was completed. The setup also permitted multi-channel stimulation with varied time intervals between stimulation pulses at the different spinal segments. This method allowed for stimulation of the cord at an optimal frequency of 40 Hz to neuromodulate activity of spinal networks below the lesion, as described previously (Shah et al., 2012, 2016; Alam et al., 2015). 90% stimulation threshold was used to avoid undesired stimulation of the cord.

Second, we tested if our setup allowed individual spiking activity within the EMG burst to trigger single ES pulses to modulate timed activity of individual EPSPs (**Figures 5B-iv–vii**), as described previously for intraspinal microstimulation (McPherson et al., 2015). The main rationale of this stimulation strategy was to modulate activity of individual spikes within a burst, presuming that each spike is an excitatory post-synaptic response generated from supraspinal descending drive or spinal activity. The stimulation pulse delivered in the specific spike window would then modulate activity of active neural circuitry to allow for the delivery of stimulation at a user-defined frequency (McPherson et al., 2015; Zareen et al., 2017). Indeed, this stimulation strategy proved to be highly customized for each trial within and between rats and yielded unique resultant (effective) frequencies of tES delivery (see section “Results”). An inter-stimulation interval of 10 ms was imposed after each stimulus pulse to prevent stimulation artifacts from retriggering stimulation (**Figure 5B-vi**).

##### Algorithm for stimulation delivery

A feature of ‘Fast Response Output’ in our software monitored incoming EMG data and let users configure output signals (analog or digital) based on threshold values of raw input voltages. All of the threshold and hysteresis checking was done in the software by the PowerPC embedded processor in the DAU, after the input signal is delivered out of the system’s Analog to Digital converter. For our purposes, each digital output was linked to 2 input channels (i.e., digital output 1 was linked to input 1 and input 2; output 2 to input 3 and 4, etc.). When the raw voltage of a monitored input crossed the set level of 0.2 V, a fixed width pulse of 0.005 s was triggered in the digital output. A hysteresis of 3% meant that the input signal needs to go above or below 0.2 V by 0.006 V before another output can be triggered again by each input pair. These Fast Response Output settings were saved in the random-access memory (RAM) in the DAU and are downloaded whenever a particular file is loaded.

### Setup for Data Collection

Application of the closed-loop system for cervical spinal cord tES was tested in two experimental conditions: A non-mobile experimental setup, tailored to deliver tES in multiple rats simultaneously during overnight activity for 7 h/day for 6 days/week for ∼3 months (**Figure 2**). The second, a mobile setup, for experiments aimed at assessment and training in a variety of rehabilitative motor tasks (**Figure 3**).

#### Non-mobile Setup for Multiple Rats

We constructed a non-mobile system that is capable of simultaneously delivering the tES for 7 h/session to multiple rats, limited mainly by the units of equipment used. In our previous experiments that involved training with ES, we have been able to train only one rat at a time because of technical limitations with our experimental setup (Shah et al., 2016). This limitation becomes exaggerated for overnight experiments where rats need to be exposed to the stimulation for long hours, because data can then be collected from only one rat at a time (Gad et al., 2013). Shown in **Figure 2** is a setup for three rats that utilizes two amplifiers, one 8-channel stimulator, a desktop and one DAU; assuming two channels of multi-site stimulation, triggered by two EMG channels, and one LED pulse per rat. Each rat is housed individually in its own cage throughout the experiment. Importantly, training with tES for all rats can be started at the same time. Data collection from video as well as EMG can be controlled by a common software source. Any errors in delivering the ES or data collection steps can be easily detected and all data are also stored to a common backup simultaneously. A standard rat cage was modified to integrate the flexible swivel connector within the rat’s cage lid that connected to the stimulation device. Three holes matched to the slip-ring manufactured screw placement were drilled into a piece of hard plastic. The slip-ring was secured to the hard plastic using three screws and nuts. The hard plastic with the slip-ring was then secured to the cage lid via bolts. A curved water bottle spout was connected to the rat’s water bottle and inserted into the bottom hole and secured. Modifying the home-cage in this way ensured that the rats could move freely in their cage while the stimulation is on. During the overnight session, rats were filmed using an IR camera to visually observe overnight cage activity concomitant with EMG recording.

#### Mobile Setup for Experiments in an Individual Rat

We scaled down the non-mobile system into a mobile system for tES delivery in an individual rat to include a single amplifier, stimulator, camera and a laptop (**Figure 3**). Although all connections of the mobile setup were similar to the non-mobile setup, a webcam was used that was controlled by a recording software that automatically synced the video feed to the EMG traces. The setup also was less cumbersome requiring minimal maintenance with cable and rat-cage usage, enough for testing or training an individual rat. We tested its functionality in a variety of portable experimental settings for motor behavioral testing in our laboratory.

### Data Collection

Data were collected in two experimental phases described below.

#### Overnight Experiments

We tested the capability of the non-mobile closed-loop stimulation system to deliver tES throughout the night in response to the EMG activity during the rat’s free activity in its home-cage. This was done in non-injured rats to test the logistics, feasibility and implementation of the 7 h protocol prior to employing it in injured rats. A full night-time session lasted for 7 h and consisted of 30 min of baseline EMG and video recording in the home cage, followed by 6 h of stimulation and recording, and a subsequent 30 min of post-stimulation recording. The deltoid, pronator or extensor muscle was chosen to trigger tES because our preliminary EMG data revealed that these muscles are the first to activate during any attempted locomotor movement of the forelimb, including isometric contractions. tES was triggered from onset of EMG burst or spiking EMG activity as described above.

We then determined if long-term closed-loop tES could be applied in the rat’s home-cage without the need for supervision in multiple rats after the injury. Seven CHx rats underwent long-term overnight exposure to tES for 7 h/day for 6 days/week for 1–3 months, beginning at 10 days post-injury. Our preliminary data show that 10 days post-injury is the time when rats with a complete hemisection at C4 have regained adequate control of their hindlimbs and trunk to move around in the cage. These rats are also most physiologically stable by day 10 after an initial insult to the rostral cervical cord. The stimulation protocols were the same for non-injured and injured rats, as descried above in *stimulation protocol*.

#### Mobile Experiments

The feasibility of the closed-loop system was then assessed for use in mobile experiments. Specifically, ES was triggered from onset of EMG activity and/or individual spiking activity (EPSPs) in the EMG burst during different motor behaviors: ladder walking, treadmill walking, rearing and grooming behavior and reaching-grasping. Rearing and grooming behaviors were chosen because these are the rat’s most naturally occurring behaviors. For ladder walking, a rat was placed on a horizontal ladder consisting of unevenly spaced rungs with cages on either end of the ladder serving as end points (Metz and Whishaw, 2009). For treadmill walking, rats were placed on a custom-built rat treadmill similar to the one used previously (Shah et al., 2013) and the speed was set to 13 cm/s. For the rearing behavior, rats were placed in a custom built acrylic cylinder that readily prompted the rearing behavior. The grooming behavior in the cylinder was obtained via natural grooming activity or grooming induced manually by placing few water drops on the rat’s head between the ears (Gensel et al., 2006). For reaching-grasping pellet retrieval task, rats were assessed on their ability to reach and grasp sugar pellets successfully as described previously (Zemmar et al., 2015; Alam et al., 2017). The rats were placed individually in an acrylic reaching and grasping chamber (18 cm × 15 cm × 31 cm) with a small slit in the front wall (3 cm × 1.5 cm). Rats were offered with a 45 mg banana-flavored sugar pellets (Bio-Serv, United States) and encouraged to reach and grasp the sugar pellet placed on the platform 1cm away from the slit with the preferred forepaw.

All sessions were recorded throughout the experiments so as to obtain EMG and video data during these functional activities. The EMG signals were filtered (band passed, 10 Hz–5 KHz) and amplified (1000×) using an analog amplifier (differential AC amplifier, AM-systems Inc., United States). The signal then was digitized at a 10 KHz sampling rate and stored on a computer using a data acquisition card (NI-DAQ; National Instruments Inc., United States) using a custom-written program. Data obtained from these experiments were set to automatically store into our lab servers for use in our current and future works.

### Data Analysis

Electromyography and video data were synchronized to match movement with corresponding EMG activity. For this, we first find the first frame where the LED sync is on (LED onset) in the video (example: 50). In the associated EMG file the LED onset corresponds to the first rise in voltage in the *LED sync* channel (example: 5 s). From a range of video frames that contain a motor event (example: reaching/grasping) (example: 500–1500), frame values are converted to values relative to the LED onset frame (example: 450–1450). The relative frame values are then converted to time values by dividing with the video’s frame rate (4.5–14.5 s for a 100 fps video). For the EMG file, all time points before the LED onset in the EMG file are cropped to create a new file. As such, the time values generated from the video then match the EMG file (the software automatically takes care of the sampling rate for the EMG file). Note that EMG signals were band-passed filtered (20–1000 Hz) for data analysis. Data were analyzed to verify if stimulation was triggered secondary to the appropriate EMG trigger. The latency to onset of stimulation was obtained as the time difference between EMG activity triggering ES and onset of stimulation.

For all data, representations of EMG bursts from the most clearly visible cycles during ladder walking, treadmill walking, reaching-grasping, grooming and rearing activities on the video were selected for data analysis. Clear motor activity was first identified on the video and corresponding EMG activity detected to show success of the closed-loop system at delivering ES triggered from onset of bursting activity for overnight experiments as well as from individual spikes for motor behaviors in the mobile setup. We watched videos for gross behavior and then the corresponding EMG activity was retrieved to study which muscle triggered the tES. All EMG analysis for this manuscript was limited to identifying the appropriate muscles for tES and testing the feasibility, reliability and validity of the closed-loop tES.

## Results

### Feasibility

In this work, we successfully assembled a functional and simple closed-loop system without the need for designing complex electrical circuit boards or electrical neuro chips. We were able to establish use of the setup for overnight experiments requiring overnight tES of the spinal cord in injured rats, possessing capabilities to function without tester supervision (**Table 1**). The system could also be implemented in a variety of experimental settings for use in motor behavioral studies.

**Table 1:** Features of the closed-loop system.

Feature	Description
Off-the-shelf parts	No requirement of complex circuit boards/chips.
Versatile and customizable	The system can be customized to receive input and provide output from/to a variety of different sensor and stimulation sources, custom tailored to individual experiments.
Expandable	We demonstrate an electronic setup for up to three animals in **Figure 2** (assuming two amplifiers, one DAU, and 1 stimulator using 2 channel EMG, stim, and LED). The system as is further expandable to more animals, if needed.
Low latency	Latency times are 0.2 ms between the EMG signal crossing threshold and the stimulation activation (**Figure 5**).
High number of inputs and outputs	The system, assuming one DAU, allows for 16 channels of input and 10 channels of output. Adding a second DAU allows for an additional 16 channels of input (no extra output channels).
Can trigger off multiple inputs separately or simultaneously	The system can be configured to initiate multi-site stimulation based off individual or simultaneous threshold activation (**Figures 6, 7**).
A/C controlled	The system is A/C powered allowing for lengthy recording times.
Application for chronic experiments	System is feasible and reliable for use in overnight stimulation studies that does not require supervision.
Portable	The system is mobile, easily transportable in a cart, making it ideal for a variety of behavioral testing and training protocols.


### Reliability

We were able to reliably implement the closed-loop system to deliver the stimulation protocol for several sessions throughout 1–3 months, a prime requirement for our future chronic training experiments (**Table 2**). Although there was no problem with the system itself, in one rat, experimentation was stopped by ∼1 month because the headplug failed. We also encountered an epidural implant failure in a couple of rats.

**Table 2:** Success of conducting night experiments using the closed-loop system over select time points.

Rat identity	Pre-injury testing and experiments (non-injured rats)								
Rat 1	✓								
Rat 2	✓								
	
	**Post-injury testing and experiments (injured rats)**								
	
	**10**	**20**	**30**	**40**	**50**	**60**	**70**	**80**	**90**
	
Rat 3	✓	✓	✓	✓	✓	✓	✓	✓	✓
Rat 4	✓	✓	✓	✓	✓	✓	✓	✓	✓
Rat 5	✓	✓	✓	✓	✓	✓	✓	NR^∗^	NR^∗^
Rat 6	✓	✓	✓	✓	✓	✓	✓	✓	✓
Rat 7	✓	✓	✓	Omnetics connector malfunction. Testing and experiments discontinued						
Rat 8	✓	✓	✓	NR^∗^	NR^∗^	NR^∗^	NR^∗^	NR^∗^	NR^∗^
Rat 9	✓	✓	✓	✓	✓	✓	✓	✓	✓



*✓ – Testing and experiments successfully performed. ^∗^NR – Non-responsive to tES due to epidural implant failure.*

The closed-loop system allowed successful tES delivery simultaneously in multiple rats during overnight activity for 7 h/day for 6 days/week for 1–3 months. We were able to start experiments for all rats at the same time, the system automatically stopped the experiments at the end of 7 h, ran the 7 h protocol homogenously for all rats, and EMG-video data were collected and saved automatically without any software system errors. The head plug connectors, cables, swivel connectors and customized home-cages functioned well without major errors throughout the experimental timeline. The cables remained viable and did not interfere with night activity, food or water access in the cage. Note that although we demonstrate feasibility of the system to deliver tES to three rats simultaneously in this work, preliminary data from our lab now show that we can expand this system to deliver tES in eight rats. Application of the closed-loop system in the mobile setup too was successful in a variety of experimental settings that involved motor assessment. In all rats, the deltoid and extensor muscle EMG activity readily triggered ES in response to some proximal arm movement and/or distal wrist movements, respectively, by day 10 post injury during the rat’s overnight motor activity in the cage. In comparison to pre-injury levels, the EMG threshold required to trigger ES increased earlier after the injury. For example, EMG threshold to trigger ES from the deltoid changed from ∼0.03 mV (pre-injury) to ∼0.15 mV (post-injury). Likewise, EMG threshold to trigger ES from the extensor changed from ∼0.05 mV (pre-injury) to ∼0.2 mV (post-injury). The increase is most likely due to elevated motoneuronal excitability states that leads to overall increase in baseline bursting EMG activity and baseline noise after the injury, a commonly observed phenomena early after a cervical SCI (Alam et al., 2017). We are, however, considering adopting more robust EMG threshold detection techniques (such as signal noise ratios) for our current experiments. Interestingly too, stimulation intensities that induced muscle twitch after the injury were generally higher for up to 3–6 weeks post injury (0.03–0.06 V pre-injury versus 0.25–1.5 V post-injury), and gradually decreased over time. The elevated stimulation intensities that were required to induce limb movement indicate that the relatively more rigid forelimb observed from elevated spasticity after a SCI necessitated greater strengths of stimulation to allow visualization of a muscle twitch (and perhaps overcome the co-contraction). Details of these observations are currently under investigation in our laboratory and beyond the scope of the current manuscript.

### Sensitivity of ES Delivery

Our system was able to detect the set EMG thresholds to deliver stimulation 100% of the times. Additionally, there were no false negatives, that is, the current was delivered only when the set threshold for that muscle was reached.

### Validity

**FIGURE 6:**
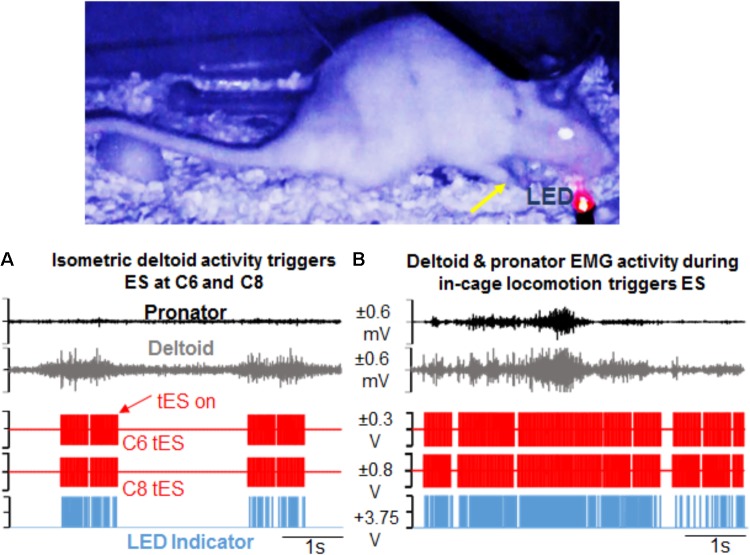
Demonstration of activity-dependent ES of the cervical cord segments triggered by pre-defined EMG signal thresholds (tES) during overnight training in the rat’s home cage. **(A)** Standstill image that corresponds to a frame in the video is synced with precise time of an ongoing forelimb EMG activity. The EMG signal corresponds to forelimb weight support (yellow arrow) in the rat’s home cage at night. Note that activity in the deltoid muscle alone triggers ES of the C6 and C8 spinal cord segments (red pulses). Stimulation occurs in response to muscle activity measured via EMG signal only at a defined threshold (tES on). With no muscle activity present, tES shuts off automatically. LED on the video blinks red when the EMG signal crosses a threshold to trigger ES and is also recorded as a pulse (blue) in the data collection software. **(B)** Shown is a similar snapshot of a video frame that corresponds to deltoid and pronator muscle EMG activity during a forelimb locomotor task in the cage. The deltoid muscle threshold keeps the ES switched on for a longer duration.

Our data confirm that for both the overnight as well as mobile setups, the closed-loop system is capable of reliably generating a pre-defined signal parameter that surpasses a user-defined threshold to reliably trigger the DAU to in turn, activate the stimulator to output the desired stimulation protocol without user input. For overnight closed-loop tES in multiple rats, the 7 h tES protocol remained constant over the 3 months. Stimulation pulse trains were consistently generated and applied over the full 6 h in response to selected EMG bursts that crossed a user-defined threshold for muscle activation. Delivery of stimulation was possible by trigger from single (**Figure 6A**) or multiple muscles (**Figure 6B**). The synchronizing LED pulse verified that the EMG signal surpassed threshold and only then generated the stimulation output. Out of the 6 h for which ES was prescribed for delivery, the total actual stimulation time ranged from 4 to 5 h per rat, depending upon the night activity of that rat. Video recording and the stimulation protocol lasted for the 6 h with half hour of baseline data collection pre-and post the tES delivery session, making it suitable for chronic implementation in injured rats. Video data generated from the IR cameras proved useful in linking patterns of EMG activity with quality of movements during free cage activity.

**FIGURE 7:**
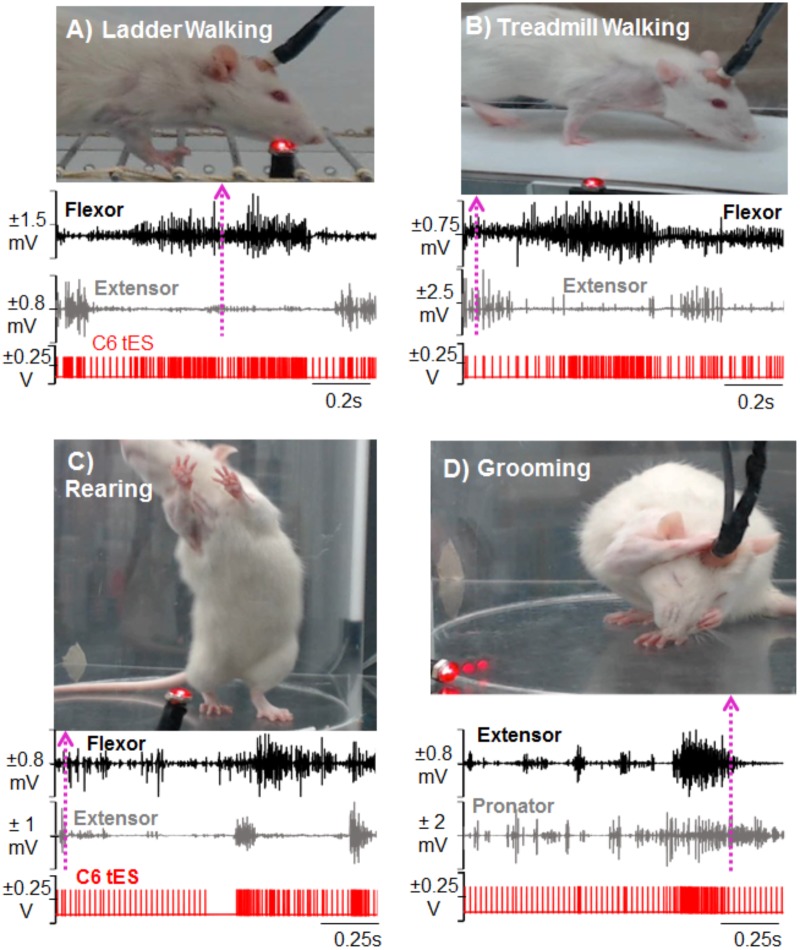
Demonstration of activity-dependent ES of the cervical cord segments triggered by pre-defined EMG signal thresholds (tES) in a mobile experimental setup designed for a multitude of motor tasks. Standstill image that corresponds to a frame in the video is synced with precise timing of an ongoing forelimb EMG activity (dotted arrow matches the frame with the time in the EMG trace). The portable closed-loop setup permitted obtaining data for ladder walking **(A)**, treadmill walking **(B)**, rearing **(C)**, and grooming **(D)** motor behaviors. Note that during each task, tES occurred following every spike in the EMG burst based upon a user-defined threshold for muscle activity.

For behavioral experiments that are done in different physical locations, transporting the mobile tES system proved beneficial since the overnight training setup is cumbersome. For ladder (**Figure 7A**) and treadmill walking (**Figure 7B**), for example, flexor and extensor muscles independently triggered stimulation. **Figures 7C,D** show the versatility of the closed-loop system in detecting spiking EMG activity to initiate stimulation while rats performed rearing and grooming behaviors.

**FIGURE 8:**
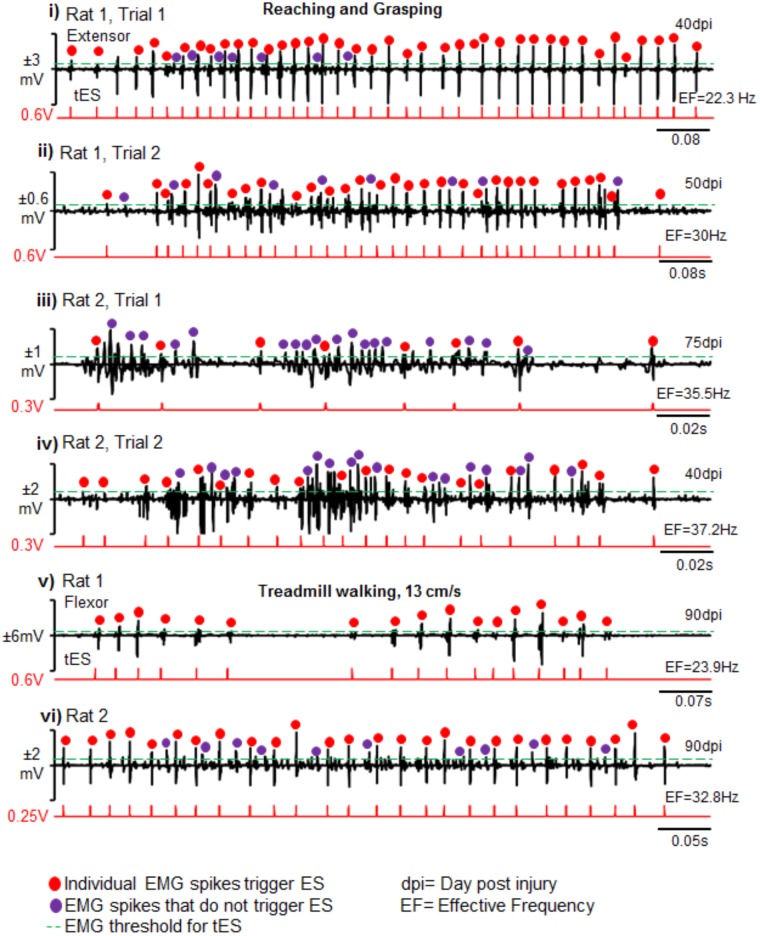
Reliability and validity of closed-loop spinal ES system. Shown are representative EMG bursts along with corresponding tES during two trials of reaching-grasping activity for Rat 1 **(i,ii)** and Rat 2 **(iii,iv)** at different days post injury (dpi). **(v,vi)** Similarly, representative EMG data from a single trial are shown from two rats (Rat 1 and Rat 2) during treadmill walking at 13 cm/s. Red dots are EMG spikes that trigger tES. Violet dots are spikes that cross the user-defined threshold, but do not trigger tES because of the preset inter-stimulation interval time. As expected, the closed-loop system consistently delivered tES in response to the EMG spikes (red dots) that crossed a tester defined threshold (green line) for a multitude of motor tasks between and within rats. Note that the resultant effective frequency (EF) directly depends upon bursting activity of the task.

Importantly too, the closed-loop system delivered tES from the onset of muscle burst or muscle spike within the burst as determined by the tester. This was consistent between rats, across trials and between behavioral conditions (representative two rats are shown across two representative time points and two testing conditions in **Figure 8**). There were no noticeable movement artifacts and interference of motor activity from cable or rat movement in any instances.

Latency to stimulation: During tES, the latency to onset of stimulation consistently occurred after crossing the user-defined muscle threshold. This was ∼0.2 ms, irrespective of whether the stimulation was triggered from onset of burst activity or by spikes within the EMG (**Figures 5-iii,v**).

### Limitations of the Closed-Loop System and Its Application for Chronic Experiments

(i) Note that the duration for which every rat receives tES is directly dependent on the voluntary activity level of rats. As such, the total treatment time using tES is variable between rats, especially after an injury. Based on our preliminary data for overnight experiments, for example, the number of hours during which tES is actually delivered to each rat ranges between 4 to 5 h per rat per session. In any case, our setup allows us to overcome this variability by customizing the training time for each rat, easily programmable into the software. (ii) Although we did not encounter any obvious problems with use of the technique itself, implementing the closed-loop system for the overnight experiments was more demanding than the mobile setup. Some complications related to this application include: (a) in rare instances, rats tend to chew on the cables. Using a shrink-wrap around the cable resolved the issue without restricting rat movement. (b) All rats were able to sustain the long durations of stimulation for the 3 months. It was however, not uncommon for rats to attempt to remove the connecting cable with their contralateral upper limb in the initial days of training. This limitation was overcome when the connection between the connecting cable and head plug was *tightened* using a thin layer of a solvent (such as nail-polish). (c) Complete failure of the head plug and/or ES wires too are other complications associated with long-term training. (d) The non-mobile setup for tES in multiple rats is cumbersome and requires committed space. Robustness of the setup is, however, ideal for chronic experiments such as those pursued in our laboratory.

## Discussion

We present herein an activity-dependent closed-loop electrical stimulation system that features an assembly of off-the-shelf components and allows for ES of the cervical spinal cord triggered from prescribed motor movements in real time. To our knowledge, this is the first study that incorporates the delivery of cervical ES triggered by EMG activity in freely moving rats after a cervical SCI. Our results show that the tES system can be successfully implemented during a variety of motor behaviors during different experimental settings. We also demonstrate the feasibility of the system for use as a long-term rehabilitative therapeutic tool for multiple rats in a chronic overnight training regimen without the need for tester supervision. Experiments in our laboratory are underway to determine if chronic intervention with tES will restore upper limb motor function from paralysis after a cervical SCI.

### Unique Features of the Developed Closed-Loop System and Experimental Setup

The closed-loop system constructed from off-the-shelf commercially available components offers wide versatility for use with both freely moving animals as well as in constrained experimental conditions. In this work, we show that delivery of cervical ES is possible for long durations (7 h/day for 6 days/week for 1–3 months) without any interruptions in a given session and without need of supervision by a person. Multiple rats can be trained at the same time, thereby greatly increasing the efficiency of experiments that utilize long-term rehabilitative protocols. Our experiments that lasted for 3 months did not suffer from any major problems in the hardware or software components of the system. Simple modifications of the home-cage with commutators and infrared capabilities when integrated with the setup, permit training rats and monitoring their night activity for long hours without tester supervision. Stimulation sequences can be pre-programmed or triggered off ongoing EMG. Additionally, depending on rodent behavior in the cage, the stimulation parameters (time of stimulation, frequency of stimulation) are continually *adapted* (in the millisecond range) throughout the treatment session in response to feedback from physiological changes (EMG activity) at any given time in the treatment session. For example, the frequency of stimulation varies continuously throughout the training period, varying between 20 and 40 Hz, depending upon the extent of motor activity by the rat (see **Figures 5B, 8**). The stimulation also stops as soon as the rat stops moving. A feedback loop from bio-signals that control the stimulation delivery parameters therefore make it a real-time adaptive (closed-loop) system (Krook-Magnuson et al., 2015; McPherson et al., 2015). This is in contrast to open-loop stimulation protocols in which the stimulus is delivered regardless of the ongoing activity and according to a pre-defined offline script. The uniqueness of this system is also the ability to use the raw EMG signal from a single and/or variety of muscles and/or motor activities within the same experimental session so as to deliver a single or multi-site stimulation pulse within a short period of time (latency of 0.2 ms). The setup can allow the use of other bio-signals (such as mechanomyography and force plate sensors) as stimulation triggers. Additionally, a streamlined platform for closed-loop experimentation using simple point electrodes makes it a promising tool for use with multi-array electrodes.

### Closed-Loop ES of the Lumbar Spinal Cord

There is growing interest in spinal ES as a means of facilitating motor output after a SCI. Most of the previous works on ES has been *open-loop* that entails delivery of continuous ES at a constant rate, not timed to EMG activity related to the task (Carhart et al., 2004; Courtine et al., 2009; Harkema et al., 2011; Shah et al., 2012; Angeli et al., 2014; Shah and Gerasimenko, 2016) for review, see (Shah and Lavrov, 2017).

In contrast to an algorithm that is embedded within our designed software, note that previous ES studies for spinal cord stimulation have employed algorithms operated by customized neuro-chips to create a closed-loop system. One of the most elegant closed-loop ES systems implemented in SCI studies integrated feedback from hindlimb kinematic activity and muscle EMG to control lower limb kinematics during continuous bipedal stepping in real time in rats spinalized at T8 (Wenger et al., 2014). This system utilized control algorithms and advanced technological platforms that interfaced feedback signals and feed-forward mechanisms to match ongoing modulated neural activity in real time. In another study involving rats that were spinalized at the thoracic cord, ES in the lumbosacral spinal cord was triggered from EMG activity of the biceps brachii muscles (Gad et al., 2012). In both these reports, ES was delivered in a setup that involved restraining the spinal rat and ES continued for as long as the rehabilitative training lasted under tester supervision. Gad et al. (2012) used an analog multiplexer controlled by a target board debugging interface (MSP-EZ430) to detect the start and stop times of EMG bursting activity in the forelimb to deliver lumbosacral stimulation. The goal of this experiment was to achieve indirect volitional control of ES (by forelimb EMG activity) to facilitate quadrupedal stepping in spinal rats. A moving window step-detection algorithm was implemented in a small microprocessor to detect the on-off EMG patterns of a single forelimb muscle that initiated and terminated lumbosacral ES during walking on a treadmill. No feedback control of the bio-signal to correct stimulation parameters were employed. The latency to stimulation from trigger was longer than 1 s (almost two step cycles) and involved signal processing (ex: FFT of raw signal) prior to stimulation delivery. This is in contrast to detection of raw spiking activity of multiple muscles that are activated during volitional motor activity in the rat’s home-cage in our setup. Our algorithm detects threshold of EMG activity with each spiking activity within 0.2 ms of spike activity onset. Our system is also set to deliver chronic periods of stimulation (for ∼7 h) independent from supervision by a tester; with the primary goal of promoting long-term plasticity in spinal neural circuitry, similar to what has been shown with intraspinal microstimulation (McPherson et al., 2015).

Collectively, our implementation of the system and algorithm is the first application of a closed-loop system for cervical spinal cord ES as well as the only one so far for chronically delivering cervical ES without tester supervision in freely moving rats during the rat’s home-cage activity.

### Novel Closed-Loop Stimulation Strategies of the Cervical Spinal Cord

Our current experiments were inspired by studies that demonstrate that triggered intraspinal microstimulation of the cervical spinal cord controlled by cortical neurons or EMG activity will evoke movements of the arm and hand in primates (Jackson et al., 2006b) and also improve hand function after a SCI in rats (McPherson et al., 2015). In the present work, we built an activity triggered ES system that allowed training rats with tES without tester supervision. The system involved utilizing EMG activity from the affected forelimb muscles to trigger stimulation of the cervical cord so as to facilitate ongoing forelimb movement patterns during routine activity in the rat’s home-cage environment.

We were able to implement two unique protocols for triggered stimulation. The first tES strategy was the delivery of a continuous 500 ms train of ES (40 Hz) triggered from the first spike of EMG bursting activity that reached a prescribed threshold value during attempted movement. Although descending supraspinal neuronal control is integral to regulate locomotion, bilateral rhythmic alternating neural activity necessary for treadmill and overground locomotion does not rely heavily on descending drive (Grillner and Zangger, 1979; Edgerton et al., 2008). We therefore tested a stimulation strategy that permitted continuous delivery of ES to target forelimb locomotion in this study. The idea that a prescribed 40 Hz frequency will globally activate locomotor networks in the cervical cord was adopted from previous reported works that demonstrate that 40 Hz can effectively facilitate pattern generated hindlimb locomotor activity after severe hindlimb paralysis [for review, see (Shah and Lavrov, 2017)]. Moreover, previous studies that have employed cervical ES (Alam et al., 2015, 2017) or cervical intraspinal microstimulation (Sunshine et al., 2013; Mondello et al., 2014) have shown success in modulating cervical networks at these frequencies.

The main feature of the tES mode was to trigger ES secondary to initiation of any attempted activity in the cage, instead of continually delivering ES even when the muscles were at rest. Our data indicate that at rest, EMG activity during tES was close to zero as is desired during the muscles’ resting phase, to eliminate unnecessary levels of excitation from the muscle. Although continuous delivery of electrical stimulation has shown improvements in a variety of physiological functions after a severe SCI in both animals and humans [for review see (Shah and Lavrov, 2017)], findings from literature demonstrate that delivering ES triggered by EMG activity is a far better stimulation strategy than continually delivering ES even during the rested no-EMG activity phases (Krook-Magnuson et al., 2015; McPherson et al., 2015). This is most likely because the excess delivery of electrical current into a relatively inactive neural circuitry zone does not necessarily result in neuromodulation that is otherwise required when ‘demanded’ of the nervous system, as during an attempted movement. This concept is supported by clinical studies in humans with a functionally complete SCI. In these works, although ES is continuously delivered during volitional attempts made by the subject, greater gains in voluntary leg movements are seen in those subjects when ES also accompanies volitional effort and intent to move (Angeli et al., 2014; Grahn et al., 2017; Rejc et al., 2017). It is reasonable to hypothesize that delivery of stimulation at a time when the circuits are ‘primed’ for activity, will produce functional benefits than when delivered without volitional attempts to move. These questions, however, need to be addressed systematically and are some of the objectives of our future experiments.

The second tES strategy presented here involved delivery of single ES pulses triggered from individual spikes of EMG activity. In this protocol, each stimulation pulse was delivered in response to spikes in forelimb EMG burst in a spike-activity dependent manner during free home-cage or reaching and grasping activity. The fundamental premise of this stimulation strategy is to amplify an individual excitatory post-synaptic response by enhancing supraspinal descending drive in a specific window to facilitate long term potentiation and modulate activity of active neural circuitry to allow for the delivery of stimulation at a user-defined frequency (McPherson et al., 2015; Zareen et al., 2017). In an *in vitro* experiment, after a cervical spinal cord hemisection in adult rats, inspiratory bursting in the tongue muscle that is used to trigger intraspinal microstimulation resulted in activation of diaphragm motor units during the inspiratory phase (Mercier et al., 2016). This paradigm evoked short-term potentiation of spontaneous inspiratory activity in the previously paralyzed hemidiaphragm (i.e., bursting that persists beyond the stimulus period). Moreover, chronic treatment with intraspinal microstimulation of the cervical spinal cord that is timed with spiking EMG activity during reaching and grasping function, greatly enhanced motor function (McPherson et al., 2015). None of the rats in the present study were exposed to any training using this protocol, but the presented data are proof of concept for implementing the EPSP triggered ES as a more refined technique to control a more precise supraspinally driven skilled motor output. Experiments in our laboratory are currently under way to determine the effects of this ES protocol on skilled reaching and grasping function in injured rats.

Note that we have not reported the benefits of this system over the use of continuous ES for the cervical cord after a cervical SCI, but this question is currently beyond the scope of the present report. Instead, the methodology for tES described here has laid the foundation for pursuing this and similar crucial research questions with use of the proposed technique.

### Overnight Activity-Dependent Chronic Stimulation

One of the major advantages of our setup has also been the ability to stimulate the spinal cord for prolonged hours through the rat’s night cycle. After severe cervical spinal cord injuries, the motor deficits are persistent (Filli et al., 2011) and therapies to enable motor function of the paralyzed limb often necessitate chronic intervention periods of motor and/or neuromodulatory strategies (Angeli et al., 2014; Grahn et al., 2017). However, intervention time for a given therapy is limited both in the standard pre-clinical and clinical setting. There is therefore a need for automation in motor therapies delivered, but in a way that integrates the subject’s intentional activity and functional capability. Moreover, as expected, the effects of stimulation training are maximum when the subject is actively engaged in training (Angeli et al., 2014; Buick et al., 2016). As such, delivering stimulation in the rat’s active (i.e.) night cycle will deliver stimulation for a longer period – each time the rat attempts to move. The delivered stimulation in turn will activate spinal circuits to further permit movements that were otherwise not possible – thereby creating a positive feedback mechanism of increased ‘self-training.’ Indeed, experiments that utilize such long-term activity-dependent stimulation during overnight activity have gained increased attention (Zimmermann and Jackson, 2014; McPherson et al., 2015) and are highly relevant for pre-clinical studies that necessitate long intervention periods for effective clinical translation.

## Concluding Remarks

In the clinical setting, the use of closed-loop systems is used for a variety of neurological disorders [see review (Krook-Magnuson et al., 2015)] including its proven use for suppressing clinical seizures in humans (Fountas et al., 2005) and treating movement disorders in patients with Parkinson’s disease (Little et al., 2013). The idea that residual proximal limb movements trigger pre-programmed stimulation to cause the paralyzed muscles to contract has proved successful for closed-loop functional electrical stimulation systems controlling limb activity in humans [for review see (Ethier and Miller, 2015)].

Given the clinical use of ES in persons with a SCI (Harkema et al., 2011; Angeli et al., 2014; Grahn et al., 2017; Rejc et al., 2017), it is not surprising that much effort is being expended in improvising on ES techniques as a training tool to restore physiological functions after a SCI. Moreover, with the extreme reliance of upper limb motor function on supraspinal pathways (Fouad et al., 2001; Ballermann and Fouad, 2006; Hurd et al., 2013), it is worthwhile developing a neuro-rehabilitative feedback based intervention that integrates spinal cord stimulation with attempted voluntary movements of the hands or legs. This is especially relevant in instances of functionally incomplete SCIs that have some descending control of movement.

With the surge in evidenced-based motor rehabilitation practice, one of the main hurdles for effective translation of neuro-rehabilitative motor therapies into the community is the limited amount of time spent by patients in rehabilitation clinics (Newitt et al., 2016). To combat this limitation, carefully designed feedback based home-therapy programs have indeed become an integral part of modern evidence-based neuro-rehabilitation practice (Buick et al., 2016; Zbogar et al., 2016). The successful development of the tES technique presented herein can operate autonomously for extended periods of time in unrestrained rats, allowing its use as a long-term therapeutic tool. The system can also be used in concurrence with motor rehabilitative training procedures to facilitate the effects of the training. It can also enable us to study the short-term as well as long-term physiological effects of incorporating electrical stimulation techniques into the nervous system. It might be worthwhile for example, to investigate if an activity-dependent closed loop ES technique in combination with rehabilitative motor training will prove effective in regaining skilled upper limb reaching and grasping function in the more clinically relevant contusion injury model. Spinal ES has received attention in the treatment of neurological damage that is beyond a SCI, such as in ameliorating motor symptoms of a Parkinson’s disease (Fuentes et al., 2009). Triggering ES during resting states (as compared to during EMG activity) is another feasible application of the closed-loop system. The system can therefore prove effective both as a supplement to existing rehabilitative motor therapies in the clinic or laboratory setting, or could be used independently as a feedback based training tool.

The developed closed-loop system can also be considered to represent a class of bidirectional neural prostheses (Jackson et al., 2006b) via a circuit that enables two-way interactions between neural activity (real-time processing of EMG activity) and external devices (such as a stimulator). Recently, elaborate computational platforms such as the Brainet (Pais-Vieira et al., 2015; Ramakrishnan et al., 2015), which allows sharing neural information between multiple study subjects to engage in a common motor behavior, have gained increased attention in the field of neuroscience. This closed-loop ES technique can be experimented for connecting several neural systems into a Brainet by combining neural signals from multiple rats dynamically and in real-time so as to enhance motor performance. EMG or spinal neural activity can be recorded from the chronically implanted epidural electrodes and analyzed in real time to be delivered to the spinal cord of all rats connected in the setup. It is our hope that the features and usability of this system will encourage researchers to capitalize on the exciting possibilities inherent in closed-loop devices for ES neuromodulation studies.

## Author Contributions

AR built the proposed technology, performed the experiments, collected data, generated figures, and wrote the manuscript. PS collected data, performed all animal training experiments, analyzed and interpreted the data, generated figures, and edited the manuscript. PKS built the proposed technology, designed the experiments, performed the surgeries, performed experiments, interpreted the data, and wrote the manuscript. All authors read, edited, and approved the final draft of the manuscript.

## Conflict of Interest Statement

The authors declare that the research was conducted in the absence of any commercial or financial relationships that could be construed as a potential conflict of interest.
